# Obstruction in the Region of the Pylorus1Read at the thirty-ninth annual meeting of the Medical Association of Central New York, at Syracuse. October 16, 1906.

**Published:** 1907-03

**Authors:** Thomas Jameson

**Affiliations:** Rochester, N. Y.; 346 West Avenue


### Obstruction in the Region of the Pylorus.[1]

By THOMAS JAMESON, M. D., Rochester, N. Y.

THE knowledge of the pathology of all abdominal lesions has been greatly aided by the observation of the surgeon who has enabled us to know what is actually going on in the body during life. How little would we know today of the different forms and stages of appendicular disease if we had to depend entirely upon post-mortem observation. We are now just beginning to learn in the same manner the changes due to pathological conditions in the region of the pylorus and are thereby

1. Read at the thirty-ninth annual meeting of the Medical Association of Central New York, at Syracuse. October 16, 1906.

enabled to judge more intelligently of the relation of symptoms to the pathological lesions causing them.

When we think of pyloric obstruction, the picture that is before one's mind is of more or less complete obstruction with a dilated stomach and so forth, but long before this stage is reached, certain symptoms develop which I wish to discuss for a few moments. Works on the diseases of the stomach divide pyloric obstruction into two divisions,—Functional and Organic. Under the head of "Functional" is spasm of the pylorus. That is a sudden, tetanic contraction of the pylorus lasting during a long period and then suddenly relaxing; affording a complete relief to the symptoms.

The most common causes of pyloric spasm are ulcer, commencing cancer or the irritation produced by the presence of gall stones or of gall bladder disease. It also occurs in hypersthenic gastritis and in some conditions associated with hyperchlorhydria. The most common symptom is gastric flatulency; the gases accumulate in the stomach and are gotten rid of by belching or by the sudden relaxation of the pylorus, allowing them to pass into the intestines or in severe cases, by vomiting. There is no distress in the stomach when it is empty.

When we consider that the pylorus is physiologically a sphincter and that its function is to protect the intestines on one side from a too rapid passage of the contents of the stomach and to protect the stomach on the other, from regurgitation of bile and intestinal contents, we realize how irritation on either side of the sphincter will produce spasmodic closure.

There is another sphincter equally important which lies in the duodenum just below the entrance of the bile and pancreatic ducts. This sphincter has been demonstrated by Ochsner, of Chicago, (Annals of Surgery, January, 1906), and has frequently been observed by him in a stage of firm contraction. The presence of this sphincter throws an important light on certain symptoms often observed in obstruction in the neighborhood of the pylorus and helps us to distinguish between spasmodic obstruction due to the presence of an ulcer or other irritation in the region of the pylorus and one in the region of the duodenum.

If bile is present in the first few mouthfuls of vomitus, it rather points to the focus of irritation being below the region of the bile ducts and is most likely due to ulcer in the duodenum, or to a stone in the ampulla of Vater.

Too often patients drift from one doctor's office to another with symptoms which point strongly to pyloric spasm and are treated for "dyspepsia," "indigestion," and "liver trouble," without once having had a thorough examination and an analysis of the stomach contents or any persistent effort being made to discern their real condition.

Let me describe an illustrative case: Mrs. L.—, aged fifty; a German woman, who has suffered for years with distress after eating, eructations of gas, occasional attacks of alimentary vomiting. No history of jaundice. She had consulted many men for relief and had been under the care of a very competent physician who has devoted considerable time to the study and treatment of stomach lesions. He had given her test breakfasts and prescribed a diet which resulted in partial relief. During one of her attacks of pyloric spasm and vomiting, she consulted Dr. George S. Burns of Rochester. He made a diagnosis of gall bladder disease and asked me to see the patient with him. When we explained to her what we thought her attacks were due to, she readily consented to an operation which the writer performed at St. Mary's Hospital, and found a large gallstone at the junction of the cystic and common ducts. It was readily removed by milking it back into the gall bladder which was drained. This patient's symptoms were so strongly suggestive of pyloric obstruction of an organic nature, that I made all preparations to do a partial gastrectomy if it were necessary.

There is another form of intermittent pyloric obstruction which is due to adhesions to surrounding organs or to the malposition of the pylorus. I had a case illustrating this about eight years ago. A young man aged 28 complained of intense distress after eating a hearty meal, often followed by severe vomiting, but if he restricted himself to a liquid diet or would only eat very light meals and eat often, he would have no distress whatever. On an exploratory incision, I found the fundus of the gall bladder firmly adhered to the greater curvature of the stomach, near the pylorus, in such a way that if the stomach was at all distended it not only caused a bending and narrowing of the pylorus, but also sharply distorted the cystic duct. Sewing the gall bladder up in place completely relieved all his symptoms.

I could mention many similar cases did time permit or if it would serve any good purpose, but here let me urge the necessity of giving our chronic stomach cases more particular attention. Have the stomach contents examined; make a careful physical examination of every patient complaining of stomach symptoms and one will be surprised how often one will come across distinct pathological lesions.

In obstruction due to organic changes in the pylorus it is important to recognise that obstruction exists in the early stages. Whether that is due to commencing cancer or to the cicatrical contraction of an old ulcer, or to that form of benign cellulo-muscular hypertrophy, which often results from chronic hypersthenic gastritis. Pyloric obstruction as a rule, develops slowly and goes through three well marked stages,—the periods of compensation, stagnation and retention.

The degree of compensation depends on the rapidity of the formation of the obstruction and the power of the muscle of the stomach wall to hypertrophy. The stomach wall, in slowly developing stenosis, may more than double the normal thickness. This is much more marked in the region of the pylorus. When we have a patient who habitually suffers from very great stomach disturbances by dietetic excesses, especially the eating of large quantities of coarse food and if that patient can eat and comfortably digest a small meal that is finely subdivided by proper preparation and thorough mastication, it is more than likely that that patient is in the first stages of pyloric obstruction.

In this stage the stomach can be felt periodically contracting during digestion. Fluids are more readily evacuated than solids. All these characteristics are absent in myasthenia with which pyloric obstruction is often confounded. The duration of this stage depends on the nature of the obstruction, being a question of years in benign obstruction or a moderate cicatrical stenosis, or of a very short time in carcinoma.

In the period of stagnation, we find the muscle of the stomach wall beginning to get tired of the effort to force the food past the obstruction. It empties itself slowly but never contains food in the morning before breakfast. There often is rebellious vomiting during this period from the irritation of the fermenting contents. Pain, sometimes very severe, is likely to be a symptom of the stagnation, although there may not be much loss of weight at this time. The third period of retention is characterized by the failure of the stomach to evacuate its contents at any time during the twenty-four hours, and by copious vomiting of accumulated foods. The symptoms due to the absorption of an insufficient quantity of nutriment and water are combined with those of gastric autointoxication. Emaciation and loss of strength, may be very great, the patient becoming cachectic, and unless relieved by operative measures death results.

The patient whose stomach I have here, was a man forty years old, who had had stomach symptoms of gradually increasing obstruction for the past four years. During that period he was under the care of several different physicians and was dosed with stomach remedies ad nauseam. During the three or four years of his sickness, he never had a physical examination, nor a test breakfast, but was told by two physicians that he had cancer of the stomach and that he could not get well. He finally came under the care of Dr. M. M. Talpin of Rochester, who made a diagnosis of pyloric obstruction and asked me to see him.

I found a man extremely thin and yet able to be up around the house, who complained of distress after eating, gas, and vomiting. He would go two days without vomiting and then vomit all he had eaten for that period, the vomitus often half-filling a slop jar. Dr. William Ewers kindly examined the stomach contents for me. The capacity of the stomach was over a gallon and the greater curvature when distended with fluid could be readily seen through the thin abdominal walls lying just above the symphysis pubis. Dr. Ewers made a diagnosis of benign stricture of the pylorus. On this being explained to the patient he readily gave his consent to an operation. At the operation, which was performed at the Rochester City Hospital, we found a firm stricture of the pylorus and also that the pyloric region was firmly attached to the pancreas. The patient's condition on the table was very poor indeed and it was considered best to do a rapid posterior gastroenterostomy for immediate relief, which was done. The patient rallied well from the operation and was able to take liquid diet on the third day; but the intestines were unable to absorb any nourishment given him, on account probably of atrophy of the intestinal lacteals and absorptive glands. He died of inanition on the eighth day after operation, practically starving to death. How much better this man's chance would have been if he had had surgical aid before the stage of emaciation.

Examination of the specimen shows no malignancy and there is no doubt that he would have recovered if the anastomosis had been done in time. There was no leaking at the line of anastomosis and no evidence of peritonitis. In obstruction due to malignant disease, the clinical picture is very similar, except that the various stages are much shorter and there is much more rapid loss of strength. Yet even in these cases partial gastrectomy if done early will save a fair proportion of them and an ever increasing proportion as the profession comes to realize that early cases can and are being cured if surgery be given a fair chance.

I saw a woman, aged seventy, in excellent general condition, who gave a history of stomach trouble commencing only last February and who had been under a physician's care in a small town almost ever since, and yet her physician had never given her any kind of an examination and finally advised her to seek advice in Rochester. On examination, I found a distinct mass in the region of the pylorus. The mass was freely movable; complete obstruction existed. The patient was unable to retain even the white of an egg. I advised an exploratory incision and the patient had the day set for the operation, when she suddenly changed her mind and I was discharged from the case.

She lived only two weeks and I have every reason to believe, starved to death.

I have not attempted to give the differential diagnosis of the various forms of obstruction in the region of the pylorus, for they can be readily found in any good textbook on the stomach, but I wish to caution against a slavish adherence to mere stomach analysis without regard to the clinical history. If one waits, for instance, until a case of carcinoma of the stomach can be fully demonstrated, by the absence of hydrochloric acid and the presence of the Boas bacillus and so forth, it will usually be too late for operative interference to be of any permanent value. I am not urging indiscriminate operating in stomach diseases; far from it: but I do say, that many cases go to an early grave or else are dragging on a miserable existence that might be cured by intelligent and competent surgery.

346 WEST AVENUE.

